# Depression and Burnout among Health Extension Workers in Ethiopia: A Cross-Sectional Study

**DOI:** 10.4314/ejhs.v33i1.7S

**Published:** 2023-04

**Authors:** Rahel Birhane, Girmay Medhin, Mekdes Demissie, Berhan Tassew, Teklemichael Gebru, Biniyam Tadesse, Mulusew G Jebena, Alula M Teklu, Negussie Deyessa

**Affiliations:** 1 CDT-Africa, College of Health Sciences, Addis Ababa University; 2 Aklilu Lemma Institute of Pathobiology, Addis Ababa University, Addis Ababa, Ethiopia; 3 MERQ Consultancy PLC, Addis Ababa; 4 Centre for Innovative Drug Development and Therapeutic Studies for Africa (CDT-Africa), College of Health Science, Addis Ababa University; 5 School of Public Health, College of Health Sciences, Addis Ababa University, Addis Ababa, Ethiopia; 6 Department of Public Health, College of Medicine and Health Sciences, Wolkite University, Wolkite, Ethiopia; 7 Department of Health Economics, Management and Policy, College of Health Sciences, Jimma University, Jimma, Ethiopia; 8 Department of Epidemiology, Institute of Health, Jimma University

**Keywords:** Depression, burnout, prevalence, predictors, health extension workers, Ethiopia, low-income setting

## Abstract

**Background:**

Depression and burnout are common among healthcare workers (HCWs) and negatively affect their well-being and the quality of the service they provide. However, the burden of depression and burnout among health extension workers (HEWs) in Ethiopia and their relationship has not been documented well.

The objective of this study was to estimate the prevalence of depression and burnout among HEWs in Ethiopia and to investigate the relationship between these conditions.

**Materials and Methods:**

We used a cross-sectional study design and collected data from 584 rural and 581 urban HEWs in Ethiopia, as part of the 2019 national health extension program assessment. The Patient Health Questionnaire (PHQ-9) and Burnout Self-Test were used to screen HEWs for depression and burnout, respectively. We used descriptive statistics to estimate the magnitude of depression and burnout, and logistic regression to examine their relationship.

**Result:**

Based on PHQ-9 cutoff scores of 10, the prevalence of major depression was 16.5% among rural and 8.9% among urban HEWs, whereas burnout risk was 39.8% among rural and 12.6% among urban HEWs. The odds of having depression among HEWs with burnout risk was relatively higher compared to those without burnout risk [For rural HEWs, the adjusted odds ratio (AOR) is 11.88 at a 95% confidence interval (CI; 5.27, 26.80), and for urban HEWs, the AOR is 11.49 at a 95% CI (5.35, 24.63)].

**Conclusion:**

The prevalence of depression and burnout is high among HEWs in Ethiopia, with a significant rural–urban difference, and burnout is a significant predictor of depression. Mental health interventions that enable prevention, early detection, and management are needed especially for rural HEWs who are in charge of preventive health service delivery for the disadvantaged rural communities.

## Introduction

In 2003, the Ethiopian Ministry of Health introduced the Health Extension Program (HEP) as a cost-effective primary care strategy to improve access to essential health promotion, disease prevention, and basic curative services (1). Health extension workers (HEWs) are assigned to deliver key essential health service packages at health posts (HPs) and in the household. To date, more than 42,000 female HEWs have been employed in rural and urban settings (2). The work of HEWs is physically and emotionally demanding, as they have high workloads, walk long distances during home visits, and deal with clients' problems, such as poverty (3, 4). Moreover, HEWs are themselves living in poverty (5). These can potentially expose HEWs to threats to their well-being, mainly depression and burnout(6).

The well-being of healthcare workers (HCWs) is crucial for a well-functioning healthcare system (7), as it can enhance HCWs productivity, efficiency, and motivation (8). Depression is a common mental disorder and a major concern among HCWs because of its association with reduced productivity, functional disability, higher suicide rates (9-13), and self-reported work insufficiency (14). It is estimated to affect 15% to 67% of HCWs in low-and middle income countries (15-25) and 20% to 50% of HCWs and medical students in Ethiopia (5, 26-28). Burnout, which is understood as a syndrome that results from prolonged work-related stress, is also common among HCWs. It affects job satisfaction (29), quality of care provision (30, 31) and productivity (32, 33). The prevalence of burnout among HCWs in sub-Saharan Africa ranges from 40% to 80% (34) and 3.8% to 36.7% (28, 35-38) in Ethiopia. Burnout is higher among HEWs than other HCWs in Ethiopia (28).

Studies indicated that there is a positive correlation between burnout and depression (17, 28, 39-41). Moreover, they reported that burnout is distinct from depression (17, 39, 42, 43). However, some argue that burnout is a consequence of depression, given the overlap between the two constructs (44). Hence, any interventions designed to improve wellbeing of HEWs should address both depression and burnout. Although many studies have consistently demonstrated high levels of depression and burnout among HCWs, no study has been conducted on the prevalence of depression and burnout among HEWs at national level in Ethiopia. Instead, studies on HEWs focused on improving job performance (45-47). Therefore, the aim of this study was to estimate the magnitude of depression and burnout among HEWs in Ethiopia and to investigate the relationship between these two conditions.

## Materials and Methods

**Context**: The study is part of the 2019 national assessment of the HEP (48), which was conducted with the aim of addressing the holistic information gap regarding the performance, determinants, and prospects of the HEP as a program and the well-being of the HEWs. Health service delivery in Ethiopia follows a three-tier system, and the lowest level is the primary health care unit (PHCU). PHCUs consist of a primary hospital, a HP, and a health center. Rural HEWs are high school graduates, and urban HEWs hold diplomas in clinical nursing. Moreover, HEWs receive a one year training on health promotion and disease prevention and the delivery of limited curative services.

**Study design**: The study employed a cross-sectional survey design. Data collection for the rural and urban HEP assessments was conducted in parallel by two teams from March to May 2019.

**Study participants**: The source population was HEWs who are engaged in the implementation of the HEP packages in rural and urban parts of Ethiopia. HEWs eligible for this study were those who worked in the target kebeles or health centers and were not on leave during the survey.

**Sample size and sampling**: This study involved 1,165 HEWs (584 from rural and 581 from urban settings). The methods used to calculate the minimum number of HEWs required to be investigated in rural and urban settings are described elsewhere.

Different sampling techniques were employed for the rural and urban HEP assessments. In the rural HEP assessment, multistage sampling technique was used to randomly sample 584 rural HEWs. Sixty-two woredas were selected randomly from the nine regions, and then six kebeles were selected from each woreda. All HEWs within the selected kebeles were approached for interviews. Conversely, for the urban HEP assessment, we interviewed all HEWs who worked in Dire Dawa city (n = 98), a random sample of those who worked in Addis Ababa (n = 381), and those from towns of the woredas that were selected for the rural HEP assessment (n = 113).

**Data collection tools**: Data were collected using structured questionnaires administered to the participants using smart phones, which were programmed using Open Data Kit (49). The questionnaires were translated into five local languages for the rural setting, back-translated into English, and pilot tested before they were used for field data collection. Patient Health Questionnaire-9 (50) and the Burnout Self-Test (51) were used to screen HEWs for depressive symptoms and burnout, respectively.

The nine-item Patient Health Questionnaire (PHQ-9) is widely used to screen for and measure the severity of depression. The items are aligned with the *Diagnostic and Statistical Manual of Mental Disorders IV* (*DSM-IV*) (50, 52). Each item asks about the frequency of depressive symptoms in the past two weeks. It has four response categories that range from 0 (*not at all*) to 3 (*nearly every day*). The PHQ-9 has shown good reliability, validity, sensitivity, and specificity in different settings (50). The Amharic version has been validated in Ethiopia, in a general hospital setting with a cutoff score of 10 (53), and in PHC settings, with a cutoff score of 5 (54). The Afan Oromo version of this instrument has also been validated in a general hospital setting (55).

Burnout level was measured using the Burnout Self-Test (51). This tool has 15 questions that cover topics such as how often the individual have been bothered by problems related to their job and the people they are working with. The items have five response categories that range from 1 (*not at all*) to 5 (*very often*). Data on demographic and work-related factors hypothesized to likely be associated with depression and burnout, such as marital status, job satisfaction, and workload, were collected using a structured questionnaire.

**Statistical analysis**: Because of varying sampling techniques used to recruit rural and urban HEWs, we analyzed the two datasets separately using STATA version 14. We took the complex sampling design in the analysis of data collected from rural settings and incorporated appropriate weights. However, such weighting was not applied while analyzing urban data.

Descriptive statistics were used to estimate the level of depression and burnout of the HEWs. The prevalence of depression was determined in three ways: using PHQ-9 cutoff scores of 5 and 10 and by obtaining a *DSM-5-*based diagnosis of major depression from the PHQ-9 scores. The requirement for a *DSM-5-*based diagnosis of major depression is one or two *DSM* core symptoms supplemented with at least three additional symptoms (56). Likewise, the prevalence of burnout was computed using a score of 32 as a cutoff on the burnout measuring scale(51).

Bivariate and multivariable logistic regressions were used to investigate the presence of a significant association between burnout and depression. In that model, a PHQ-9 score of more than 10 was considered a dependent variable and burnout (a score above vs. below 32) as an independent variable of interest. Relevant socio-demographic and work-related characteristics were considered as potential confounding variables. To allow for the clustered nature of the data collected from rural HEWs, the analysis was conducted using multilevel logistic regression. Three-level models were used with kebeles and woredas nested within regions. We also calculated the Pearson correlation coefficient to examine the strength of the linear relationship between depression and burnout. A P-value of less than 0.05 was used as an indication of statistical significance.

**Quality assurance**: Several measures were taken to ensure the quality of the data. These include using standard questions, pre-testing the instruments before field data collection, providing extensive training to the data collectors and supervisors, checking the completeness of the collected data, and conducting a quality recheck after data collection.

**Ethical considerations**: Ethical approval was obtained from the Ethiopian Public Health Institute (Ref. EPHI-IRB-151-2018). Verbal informed consent was obtained from all participants. The collected data was anonymized and stored on password-protected computers.

## Results

**Socio-demographic characteristics**: The background characteristics of study participants are summarized in Table 1. A total of 584 rural and 581 urban HEWs were included in the study. The mean age of HEWs was 27.1 (SE = 0.27) years in rural and 28.73 (SE = 0.17) years in urban settings. More than half of the study participants were married (66.1%) and in the age range of 25–34 (69 %). Most were not pregnant (91.4%) during the survey, 41.0% did not have children, and 45.8% had at most two children.

**Table 1:** Socio-demographic and work-related characteristics of study participants

Characteristics		Rural HEWs (n = 584)		Urban HEWs (n = 581)		All HEWs	

		Number	Weighted %	Number	Percent	Number	Percent
Age	<25	189	22.3	74	12.9	263	23
	25–34	361	72.5	436	76.0	797	69
	>35	34	5.2	64	11.2	98	9
	Mean ± SD	27.1 (0.27)		28.3 (4.1)		27.6 (4.6)	
Marital status	Married	399	72.2	368	63.8	767	66.1
	Single	159	24.2	189	32.8	348	30.0
	Divorced/widowed/separated	26	3.6	20	3.5	46	4.0
Number of children	0	216	31.9	262	45.1	478	41.0
	≤2	271	52.1	263	45.3	534	45.8
	>2	97	16.0	56	9.6	153	13.1
Pregnant	Yes	56	7.5	42	7.3	98	8.5
	No	528	92.5	532	92.4	1,060	91.4
Level of education as CHW	Level 3 or lower	345	51.6	0	0.0	345	51.6
	Level 4	239	48.4	0	0.0	239	48.4
	Level 4 diploma	0	0.0	476	83.2	476	83.2
	Level 5 degree	0	0.0	96	16.8	96	16.8
COC	Certified	332	57.3	323	55.7	655	56.3
	Not certified	117	42.7	186	32.1	303	26.0
Means of transportation to work	By transportation	115	62.3	359	76.9	474	72.1
	On foot	75	37.7	108	23.1	183	27.9
Years of service as HEW*	<5	223	24.5	-	-	-	-
	5–10	189	32.5	-	-	-	-
	>10	172	43.0	-	-	-	-
Working hours per week	<40	519	86.0	574	99.0	1093	93.9
	>40	65	14.0	6	1.0	71	6.1
Intention to leave	Yes	115	21.3	164	28.6	279	27.6
	No	324	78.4	407	71.0	731	72.4
Overall job satisfaction	Dissatisfied	243	43.2	242	43.5	485	42.5
	Satisfied	341	56.8	314	56.5	655	57.5

HEW: Health extension worker, COC: certificate of competency

* Years of service as HEW is missing for all urban HEWs

Almost half of the rural HEWs had a qualification of Level 3 or lower (51.6%), and most of the urban HEWs had Level 4 qualifications (83.2%). In terms of work-related characteristics, 75.0% of the rural HEWs had worked as a HEW for more than five years, and 86.0% were working less than 40 hours per week. Likewise, most urban HEWs worked less than 40 hours per week (99.0%). More than half of the rural HEWs (56.8%) and 56.5% of the urban HEWs reported that they were satisfied with their job. About one quarter of the HEWs (21.3% of the rural and 28.6% of the urban HEWs) were looking for another job during the survey. On average, rural HEWs took 49 minutes (SD = 48.94) to travel from their home to the HP, and urban HEWs took 33.25 minutes (SD = 25.24) to travel from home to their workplace.

**Prevalence of depression**: Depression was higher among the rural HEWs than among the urban HEWs. Using 5 as a cutoff score of the PHQ-9, 43.1% (95% CI: 35.6, 51.0) of the rural and 36.7% (95% CI: 32.7, 40.8) of the urban HEWs had depression. Likewise, using 10 as a PHQ-9 cutoff score, 16.5% (95% CI: 11.2, 23.8) of the rural HEWs and 8.9% (95% CI: 6.7, 11.5) of the urban HEWs had major depression. Similarly, using *DSM-5*-related diagnostic criteria, the prevalence of major depression was 5.9% (95% CI: 3.1, 11.2) among rural HEWs and 3.7% (95% CI: 2.3, 5.6) among urban HEWs.

**Figure 1:**
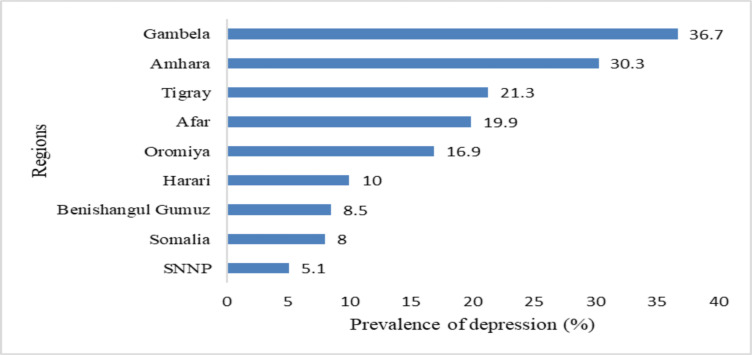
Prevalence of major depression among rural HEWs in Ethiopia, stratified by region

The magnitude of depression among rural HEWs was higher in the Gambella region (36.7%), followed by Amhara (30.3%) and Tigray (21.3%; Figure 1). Similarly, it was higher among those who work in agrarian than in pastoral settings (17.5% vs. 6.8%). Among urban HEWs, the prevalence of depression was higher in other urban regions than Addis Ababa city and Dire Dawa (8.1% in Addis Ababa, 6.9% in Dire Dawa, and 13.5% in other urban areas).

**Prevalence of burnout**: Overall prevalence of burnout risk among HEWs was 39.8% (95% CI: 32.6, 47.5) in rural areas and 12.6 % (CI: 10.0, 15.5) in urban areas. The prevalence of burnout risk among rural HEWs was higher in the Gambela region (66.7%), followed by Tigray (57.1%) and Amhara (54.7%). Likewise, it was higher among those who work in agrarian than pastoral settings (43.7% vs. 18.8%). Among urban HEWs, the magnitude of burnout risk was higher in other urban regions than Addis Ababa and Dire Dawa (42.5% in other urban regions, 5.8% in Addis Ababa, and 3.4% in Dire Dawa).

**Relationship between depression and burnout**: The results of the bivariate and multivariable logistic regressions are summarized in Tables 2, 3 and 4. In both the crude and adjusted logistic regression models, there was a significant association between depression and burnout among HEWs. The odds of having depression among HEWs with burnout risk was approximately 12 times higher compared to HEWs without burnout risk: in rural areas, AOR is 11.9 at a 95% CI (5.3, 26.8), and in urban areas, AOR is 11.5 at a 95% CI (5.3, 24.6). Except job satisfaction among HEWs in urban areas (AOR: 3.43, 95% CI: 1.60, 7.37), none of the demographic characteristics were significantly associated with depression in the crude or adjusted model (Tables 2, 3 and 4).

**Table 2:** Multivariable associations of depression and background characteristics of HEWs in Ethiopia

Characteristics	Rural HEWs (n = 584)	Urban HEWs (n = 581)

	AOR (95% CI)	AOR (95% CI)
Age		
18–24		1
25–34	0.75 (0.27, 2.06)	1.82 (0.47, 7.00)
Above 35	0.68 (0.08, 5.81)	1.21 (0.19, 7.66)
Marital status		
Single		1
Married		0.43 (0.14, 1.33)
Divorced/widowed/separated		0.68 (0.09, 4.77)
Number of children		
0	1	1
≤2	1.63 (0.69, 3.84)	1.44 (0.48, 4.28)
>2	1.37 (0.20, 6.76)	0.68 (0.11, 4.33)
Pregnant		
No		1
Yes		2.81 (0.84, 9.37)
Level of education		
Level 3 or lower		
Level 4		
Level 4 diploma		0.67 (0.29, 1.57)
Level 5 degree		1
Years of service as HEW		
<5 years	1	
5–10 years	1.12 (0.46, 2.70)	
>10 years	1.27 (0.51, 3.19)	
Overall job satisfaction		
Dissatisfied	2.17 (0.87, 5.43)	3.43 (1.60, 7.37)
Satisfied	1	

*Indicates the variables are significant (p < 0.05); CHW: community health worker; COR: crude odds ratio; PHQ: Patient Health Questionnaire; For both rural and urban HEWs, it is adjusted for burnout risk

**Table 3:** Association of depression and burnout among HEWs working in Ethiopia (n = 1,162)

Burnout risk	Major depression (Scored 10+ on PHQ-9)			

	Yes N (%)	No N (%)	COR (95% CI)	AOR (95% CI)
Rural HEWs (n = 584)				
Yes	77 (35.1)	136 (64.9)	12.56 (5.43, 7.12)*	11.88 (5.27, 26.80)*^†^
No	16 (4.3)	355 (95.7)	1	1
Urban HEWs (n=581)				
Yes	26 (52)	39 (7.6)	13.19 (6.93, 5.11)	11.49 (5.35, 24.63)*^‡^
No	24 (48)	475 (92.4)	1	1

* Indicates the variables are significant (p <0.05); HEW: Health extension worker, COR: crude odds ratio, AOR: adjusted odds ratio

† For rural HEWs, it is adjusted for age, service year, number of children, and overall job satisfaction

‡ For urban HEWs, it is adjusted for age, marital status, pregnancy, number of children, level of education and overall job satisfaction

**Table 4:** Bivariate associations of depression and background characteristics of HEWs in Ethiopia

Characteristics	Rural HEWs (n = 584)		Urban HEWs (n = 581)	

	MDD Yes N (%)	Crude OR (95% CI)	MDD N (%)	Crude OR (95% CI)
Age				
18–24	18 (11.0)	1	3 (4.4)	1
25–34	69 (18.3)	1.80 (0.81, 4.01)	42 (9.8)	2.35 (0.71, 7.83)
Above 35	6 (16.2)	1.56 (0.33, 7.30)	3 (4.8)	1.10 (0.21, 5.67)
Marital status				
Single	22 (15.8)	1	20 (10.8)	1
Married	66 (16.5)	1.05 (0.54, 2.06)	27 (7.6)	0.68 (0.37, 1.25)
Divorced/widowed/separated	5 (22.9)	1.58 (0.52, 4.84)	2 (10)	0.92 (0.20, 4.24)
Number of children				
0	27 (12.4)	1	26 (10.2)	1
≤2	49 (19.6)	1.72 (0.94, 3.17)	22 (8.6)	0.82 (0.45, 1.49)
>2	17 (14.8)	1.22 (0.39, 3.88)	2 (3.8)	0.35 (0.08, 1.53)
Pregnant				
No	85 (17.1)	1	42 (8.0)	1
Yes	8 (10.3)	0.56 (0.16, 1.99)	6 (15.8)	2.14 (0.85, 5.40)
Level of education				
Level 3 or lower	42 (16.4)	1	0 (0)	0
Level 4	51 (16.7)	1.02 (0.65, 1.61)	0 (0)	0
Level 4 diploma	0 (0)	0	37 (8.0)	0.59 (0.29, 1.19)
Level 5 degree	0 (0)	0	12 (12.8)	1
COC				
Certified	52 (18.0)	1	30 (9.6)	1
Not certified	41 (14.6)	0.78 (0.39, 1.54)	20 (8.0)	0.83 (0.46, 1.49)
Years of service as CHW				
<5 years	25 (11.2)	1	0 (0)	0 (0)
5–10 years	29 (15.9)	1.50 (0.62, 3.63)	0 (0)	0 (0)
>10 years	39 (20.1)	1.99 (0.88, 4.49)	0 (0)	0 (0)
Working hours per week				
<40	84 (15.6)	1	48 (8.6)	1
>40	9 (22.7)	1.59 (0.49, 5.20)	1 (16.7)	2.12 (0.24, 18.52)
Overall job satisfaction				
Dissatisfied	59 (21.5)	1.87 (0.89, 3.89)	34 (14.3)	3.48 (1.82, 6.64)*
Satisfied	34 (12.8)	1	14 (4.6)	1
Residence				
Same kebele	60 (15.8)	1	33 (9.9)	1
Different kebele	33 (17.9)	1.16 (0.46, 2.89)	16 (7.2)	0.38, 1.32)

* Indicates the variables are significant (p < 0.05); CHW: community health worker; COC: certificate of competency; COR: crude odds ratio; PHQ: Patient Health Questionnaire

## Discussion

This is the first study that has used national assessment data to estimate the magnitude of depression and burnout and their relationship among HEWs in Ethiopia. The findings show that depression is high among HEWs and positively associated with burnout. Moreover, depression is significantly and inversely associated with the job satisfaction of urban HEWs, but not significantly associated with the same factor among rural HEWs

The prevalence of depression among HEWs in this study is higher compared to the general population in Ethiopia, which has been reported as 9.1% (57). However, it is much lower compared to what has been reported among community health volunteers in rural Ethiopia: Women's Development Army leaders (37%) and One-to-Five leaders (26%) (5). In contrast to HEWs, those community leaders are not paid as HEWs, have low educational status, and have an increased risk of facing stressful life events such as food insecurity (5). The study also used a different assessment tool (WHO Self-Reporting Questionnaire), which might partly explain the observed difference in the magnitude of depression.

The prevalence of depression in our study is lower compared to other HCWs working in a teaching hospital in Ethiopia (26) and PHC workers in Southern Ethiopia (43). In addition to different study populations that might experience different stressors, the difference in magnitude of depression could also be attributed to using different screening instruments, such as the Depression Anxiety Stress Scale 21 (26), and the use of a lower cutoff point in the PHQ-9 (28). The prevalence of depression among HEWs in our study is similar to studies conducted in Nigeria and Brazil (15, 16, 19) or lower than findings reported among HCWs in lower and middle-income countries (17, 23-25). The variations could be due to measuring mental disorder that are not specific to depression (23-25), or use the of a lower cut-off point in the PHQ-9 (17).

In the current study, the overall prevalence of burnout among rural and urban HEWs was 36.5% and 12.6 %, respectively, and these figures are within the range of previously reported findings (28, 35, 37, 38). Likewise, it is similar to some other studies conducted among HCWs in lower and middle-income countries which range from 17% to 34.5% (23-25). On the contrary, it is much higher compared to burnout figures reported among HCWs(3.8%) in Southern Ethiopia (28), which might partly be explained by the difference in assessment tools. However, it is relatively lower compared to findings reported in a recent systematic review of burnout among HCWs in sub-Saharan Africa, which ranges from 40% to 80% (34). This could be partly explained by the working environment of our study participants, the nature of the work, and the problems with which they are dealing. HEWs work more in the community and on prevention aspects of illness, but other HCWs work in health facilities and are more engaged in patient care, exposing them to more stressful life events.

Burnout was significantly associated with depression, which is consistent with findings elsewhere (17, 28, 39, 40, 43, 58). Moreover, our study indicates that there is moderate correlation between depression and burnout (r = 0.68), which suggests that the two constructs have common elements yet are different. This finding is in line with some studies (23,44,45) but it is different from studies which reported that depression and burnout are not distinct (44, 59). Depression and burnout share some common symptoms, such as fatigue, hopelessness, and frustration, and also share a common biological basis (60). All our study participants are women, and female gender is associated with depression (19, 26, 61) and burnout (29, 34). This might partly explain the observed relationship between the two conditions.

In our study, the prevalence of depression and burnout among rural HEWs is twofold and threefold, respectively, compared to urban HEWs. The rural setting is more physically demanding and has several stresses lacking in the urban setting. Additionally, most urban HEWs in our study were nurses educated to diploma level, whereas rural HEWs had completed Grade 10 and had trained in HEP for one year. These differences in working environment and levels of education might have contributed to the higher levels of depression and burnout among rural HEWs.

Similarly, in our study, depression and burnout were more prevalent among HEWs in Gambela, Amhara and Tigray regions and among HEWs working in agrarian than in pastoralist areas. Absence of HPs in some parts of Gambela region, in particular, forced HEWs to work from their home or use health centers in distance areas. This may contribute to the higher magnitude of depression and burnout among HEWs in the region. Moreover, the geography of the majority of Tigray and Amhara regions are known for being inaccessible that can make HEWs work life difficult which in turn increases the risk of depression and burnout. More than half of the HEWs who work in those regions reported dissatisfaction with their job. The dissatisfaction might also result in the higher depression and burnout. Low education and training opportunities and disparities in benefit packages between different regions and agrarian and pastoralist settings might also explain the higher magnitude. Similarly, depression and burnout were more prevalent among HEWs who work in urban areas of other regions than those in Addis Ababa and Dire Dawa cities. The possible reason for this finding might be related to differences in benefits packages between the city administrations and other regions, and availability of professional advancement opportunities. The higher levels of depression and burnout may affect not only the HEWs but also the quality and quantity of the services they provide. Thus, attention should be paid to HEWs' overall well-being, and interventions that could improve their well-being.

To our knowledge, this is the first comprehensive national assessment of depression and burnout among rural and urban HEWs in Ethiopia. We have used weights and considered the complex sampling design during the analysis of the data from rural HEWs. This helped to reduce the risk of random errors that arises due to clustering. Nevertheless, the study is not without limitations. The instrument used to assess burnout, the Self-Burnout Test, has not been validated in our setting. Nevertheless, the instrument has shown good internal consistency among our study participants. Cornbrach's alpha was 0.92 among rural HEWs and 0.93 among urban HEWs. The other limitation is the use of a cross-sectional study design, which did not allow us to make statements about the causality and temporality of depression and burnout. This warrants further longitudinal studies.

In conclusion, depression and burnout are high among HEWs in Ethiopia and more pronounced among rural HEWs. This finding is concerning and requires interventions that enable prevention, early detection, and management to improve the well-being of HEWs. Any investment in HEWs is likely to bring returns through the provision of quality services to the community. A strategy should be in place on how HEWs can benefit from the integrated mental health services in PHCUs in Ethiopia. The high prevalence of depression and burnout among rural HEWs also indicates the need to give special attention and priority to rural HEWs. This study further shows that burnout is a strong predictor of depression. Further studies are needed to explore the impact of depression and burnout on work performance and on the quality of the services HEWs provide to their clients.

## References

[1] FMOH (2008). Health Sector Development Plan, 2005/6-2010/11, Mid-Term Review.

[2] Assefa Y, Gelaw YA, Hill PS, Taye BW, Van Damme W (2019). Community health extension program of Ethiopia, 2003–2018: successes and challenges toward universal coverage for primary healthcare services. Glob Health.

[3] Banteyerga H (2011). Ethiopia's health extension program: improving health through community involvement. MEDICC review.

[4] Bekele A, Kefale M, Tadesse M (2008). Preliminary assessment of the implementation of the health services extension program: the case of southern Ethiopia. Ethiop J Health Dev.

[5] Maes K, Closser S, Tesfaye Y, Gilbert Y, Abesha R (2018). Volunteers in Ethiopia's women's development army are more deprived and distressed than their neighbors: cross-sectional survey data from rural Ethiopia. BMC Public Health.

[6] Ridley M, Rao G, Schilbach F, Patel V (2020). Poverty, depression, and anxiety: Causal evidence and mechanisms. Science.

[7] World Health Organization (2010). Monitoring the building blocks of health systems: a handbook of indicators and their measurement strategies.

[8] Alhassan RK, Spieker N, van Ostenberg P, Ogink A, Nketiah-Amponsah E, de Wit TFR (2013). Association between health worker motivation and healthcare quality efforts in Ghana. Hum Resour Health.

[9] Cuijpers P, Smit F (2002). Excess mortality in depression: a meta-analysis of community studies. J Affect Disord.

[10] Adler DA, McLaughlin TJ, Rogers WH, Chang H, Lapitsky L, Lerner D (2006). Job performance deficits due to depression. Am J Psychiatry.

[11] Gilmour H, Patten SB (2007). Depression and work impairment. Health Rep.

[12] Ivanova JI, Birnbaum HG, Kidolezi Y, Subramanian G, Khan SA, Stensland MD (2010). Direct and indirect costs of employees with treatment-resistant and non-treatment-resistant major depressive disorder. Curr Med Res Opin.

[13] Cocker F, Sanderson K, LaMontagne AD (2017). Estimating the Economic Benefits of Eliminating Job Strain as a Risk Factor for Depression. J Occup Environ Med.

[14] Ruitenburg MM, Frings-Dresen MH, Sluiter JK (2012). The prevalence of common mental disorders among hospital physicians and their association with self-reported work ability: a cross-sectional study. BMC Health Serv Res.

[15] Aguocha Gu, Onyeama GM, Bakare MO (2015). Prevalence of Depression among Resident Doctors in a Teaching Hospital, South East Nigeria. Int J Clin Psychiatry.

[16] Obi IE, Aniebue PN, Okonkwo K, Okeke TA, Ugwunna N (2015). Prevalence of depression among health workers in Enugu, South East Nigeria. Niger J Clin Pract.

[17] Njim T (2019). Burnout as a correlate of depression among medical students in Cameroon: a cross-sectional study. J Clin Med.

[18] Ge C, Fu J, Chang Y, Wang L (2011). Factors associated with job satisfaction among Chinese community health workers: a cross-sectional study. BMC Public Health.

[19] Gherardi-Donato ES, Cardoso L, Teixeira CAB, Pereira Sde S, Reisdorfer E (2015). Association between depression and work stress in nursing professionals with technical education level 1. Rev Lat Am Enfermagem.

[20] Gong Y, Han T, Chen W, Dib HH, Yang G, Zhuang R (2014). Prevalence of anxiety and depressive symptoms and related risk factors among physicians in China: a cross-sectional study. PLoS One.

[21] Gong Y, Han T, Yin X, Yang G, Zhuang R, Chen Y (2014). Prevalence of depressive symptoms and work-related risk factors among nurses in public hospitals in southern China: A cross-sectional study. Sci Rep.

[22] Grover S, Sahoo S, Bhalla A, Avasthi A (2018). Psychological problems and burnout among medical professionals of a tertiary care hospital of North India: A cross-sectional study. Indian J Psychiatry.

[23] Malakouti SK, Nojomi M, Salehi M, Bijari B (2011). Job stress and burnout syndrome in a sample of rural health workers, behvarzes, in tehran, iran. Iran J Psychiatry.

[24] Bijari B, Abassi A (2016). Prevalence of Burnout Syndrome and Associated Factors Among Rural Health Workers (Behvarzes) in South Khorasan. Iran Red Crescent Med J.

[25] Silva ATCd, Menezes PR (2008). Burnout syndrome and common mental disorders among community-based health agents. Revista de saúde pública.

[26] Yeshaw Y, Mossie A (2017). Depression, anxiety, stress, and their associated factors among Jimma University staff, Jimma, Southwest Ethiopia, 2016: a cross-sectional study. Neuropsychiatr Dis Treat.

[27] Kebede MA, Anbessie B, Ayano G (2019). Prevalence and predictors of depression and anxiety among medical students in Addis Ababa, Ethiopia. Int J Ment Health Syst.

[28] Selamu M, Hanlon C, Medhin G, Thornicroft G, Fekadu A (2019). Burnout among primary healthcare workers during implementation of integrated mental healthcare in rural Ethiopia: a cohort study. Hum Resour Health.

[29] Rabatin J, Williams E, Baier Manwell L, Schwartz MD, Brown RL, Linzer M (2016). Predictors and Outcomes of Burnout in Primary Care Physicians. J Prim Care Community Health.

[30] Poghosyan L, Clarke SP, Finlayson M, Aiken LH (2010). Nurse Burnout and Quality of Care: Cross-National Investigation in Six Countries. Res Nurs Health.

[31] Dewa CS, Loong D, Bonato S, Trojanowski L (2017). The relationship between physician burnout and quality of healthcare in terms of safety and acceptability: a systematic review. BMJ Open.

[32] Dewa CS, Loong D, Bonato S, Thanh NX, Jacobs P (2014). How does burnout affect physician productivity? A systematic literature review. BMC Health Serv Res.

[33] Dyrbye LN, Shanafelt TD, Johnson PO, Johnson LA, Satele D, West CP (2019). A cross-sectional study exploring the relationship between burnout, absenteeism, and job performance among American nurses. BMC Nurs.

[34] Dubale BW, Friedman LE, Chemali Z, Denninger JW, Mehta DH, Alem A (2019). Systematic review of burnout among healthcare providers in sub-Saharan Africa.

[35] Biksegn A, Kenfe T, Matiwos S, Eshetu G (2016). Burnout Status at Work among Health Care Professionals in aTertiary Hospital. Ethiop J Health Sci.

[36] Lrago T, Asefa F, Yitbarek K (2018). Physicians' Burnout and Factors in Southern Ethiopia Affecting It. Ethiop J Health Sci.

[37] Bhagavathula AS, Abegaz TM, Belachew SA, Gebreyohannes EA, Gebresillassie BM, Chattu VK (2018). Prevalence of burnout syndrome among health-care professionals working at Gondar University Hospital, Ethiopia. J Educ Health Promot.

[38] Haile YG, Senkute AL, Alemu BT, Bedane DM, Kebede KB (2019). Prevalence and associated factors of burnout among Debre Berhan University medical students: a cross-sectional study. BMC Med Educ.

[39] Koutsimani P, Anthony M, Georganta K (2019). The relationship between burnout, depression and anxiety: A systematic review and meta-analysis. Front Psychol.

[40] Al-Alawi M, Al-Sinawi H, Al-Qubtan A, Al-Lawati J, Al-Habsi A, Al-Shuraiqi M (2019). Prevalence and determinants of burnout syndrome and depression among medical students at Sultan Qaboos University: a cross-sectional analytical study from Oman. Arch Environ Occup Health.

[41] Toker S, Biron M (2012). Job burnout and depression: unraveling their temporal relationship and considering the role of physical activity. J Appl Psychol.

[42] Bakker AB, Schaufeli WB, Demerouti E, Janssen PP, Van Der Hulst R, Brouwer J (2000). Using equity theory to examine the difference between burnout and depression.

[43] Creedy D, Sidebotham M, Gamble J, Pallant J, Fenwick J (2017). Prevalence of burnout, depression, anxiety and stress in Australian midwives: a cross-sectional survey. BMC Pregnancy Childbirth.

[44] Schonfeld IS, Bianchi R (2016). Burnout and depression: two entities or one?. J Clin Psychol.

[45] Kok MC, Broerse JE, Theobald S, Ormel H, Dieleman M, Taegtmeyer M (2017). Performance of community health workers: situating their intermediary position within complex adaptive health systems. Hum Resour Health.

[46] Alhassan RK, Spieker N, van Ostenberg P, Ogink A, Nketiah-Amponsah E, de Wit TFR (2013). Association between health worker motivation and healthcare quality efforts in Ghana. Human resources for health.

[47] McCord GC, Liu A, Singh P (2013). Deployment of community health workers across rural sub-Saharan Africa: financial considerations and operational assumptions. Bull World Health Organ.

[48] Yibeltal KA, Girmay M, Alula MT (2023). National assessment of the Health Extension Program in Ethiopia: study Study protocol and high-level findings. Ethiop J Health Sci.

[49] Hartung C, Lerer A, Anokwa Y, Tseng C, Brunette W, Borriello G (2010). Open data kit: tools to build information services for developing regions. roc 4th ACM Int Conf Nanoscale Comput Commun NanoCom 2017.

[50] Kroenke K, Spitzer RL, Williams JB (2001). The PHQ-9: validity of a brief depression severity measure. J Gen Intern Med.

[51] MindTools (2019). Essential skills for an excellent career.Burnout Self-Test.

[52] Spitzer R, Williams J, Kroenke K (2014). Test Review: Patient Health Questionnaire–9 (PHQ-9). Rehabil Couns Bull.

[53] Gelaye B, Williams M, Lemma S, Deyessa N, Bahretibeb Y, Shibre T (2013). Validity of the Patient Health Questionnaire-9 for Depression Screening and Diagnosis in East Africa. Psychiatry Research.

[54] Hanlon C, Medhin G, Selamu M, Breuer E, Worku B, Hailemariam M (2015). Validity of brief screening questionnaires to detect depression in primary care in Ethiopia. J Affect Disord.

[55] Woldetensay YK, Belachew T, Tesfaye M, Spielman K, Biesalski HK, Kantelhardt EJ (2018). Validation of the Patient Health Questionnaire (PHQ-9) as a screening tool for depression in pregnant women: Afaan Oromo version. PLoS One.

[56] American Psychiatric Association (2013). Diagnostic and statistical manual of mental disorders (DSM-5®).

[57] Hailemariam S, Tessema F, Asefa M, Tadesse H, Tenkolu G (2012). The prevalence of depression and associated factors in Ethiopia: findings from the National Health Survey. Int J Ment Health Syst.

[58] Fitzpatrick O, Biesma R, Conroy RM, McGarvey A (2019). Prevalence and relationship between burnout and depression in our future doctors: a cross-sectional study in a cohort of preclinical and clinical medical students in Ireland. BMJ Open.

[59] Wurm W, Vogel K, Holl A, Ebner C, Bayer D, Mörkl S (2016). Depression-burnout overlap in physicians. PLoS One.

[60] Bianchi R, Schonfeld IS, Laurent E (2015). Burnout–depression overlap: A review. Clin Psychol Rev.

[61] Naidu K, Torline JR (2019). Depressive symptoms and associated factors in medical interns at a tertiary hospital. S Afr J Psychiatr.

